# A swin transformer-based hybrid reconstruction discriminative network for image anomaly detection

**DOI:** 10.1038/s41598-025-10303-8

**Published:** 2025-09-30

**Authors:** Jin Jin, Yuanping Xu, Hui He, Feng Gao, Wenhan Zeng, Weiye Wang, Benjun Guo, Zhijie Xu

**Affiliations:** 1 School of Software Engineering, Chengdu University of Information Technology, Chengdu, 610225 China; 2 School of Computing and Engineering, University of Huddersfield, Huddersfield, HD1 3DH UK; 3 School of Advanced Technology, Xi’an Jiaotong-Liverpool University, Suzhou, 215123 China

**Keywords:** Anomaly Detection, Swin Transformer, Unet, Image Reconstruction, Engineering, Mathematics and computing

## Abstract

Industrial anomaly detection algorithms based on Convolutional Neural Networks (CNN) often struggle with identifying small anomaly regions and maintaining robust performance in noisy industrial environments. To address these limitations, this paper proposes the Swin Transformer-Based Hybrid Reconstruction Discriminative Network (SRDAD), which combines the global context modeling capabilities of Swin Transformer with complementary reconstruction and discrimination approaches. Our approach introduces three key contributions: a natural anomaly image generation module that produces diverse simulated anomalies resembling real-world defects; a Swin-Unet based reconstruction subnetwork with enhanced residual and pooling modules for accurate normal image reconstruction, utilizing hierarchical window attention mechanisms, and an anomaly contrast discrimination subnetwork based on convolutional Unet that enables end-to-end detection and localization through contrastive learning. This hybrid approach combines reconstruction and discrimination paradigms to improve anomaly detection performance. Experimental results on the industrial dataset MVTec AD demonstrate that SRDAD achieves competitive performance, with improvements of 0.6% in detection accuracy and 0.7% in localization precision. The method demonstrates improved performance in detecting small anomalies and maintaining performance in noisy environments, highlighting its potential for industrial applications.

## Introduction

With the increasing demands for quality control in manufacturing, defective products that reach consumers can result in significant economic losses and safety concerns^1^. Industrial anomaly detection serves as a critical component in modern quality control systems. This technology requires the accurate identification of defective products and the precise location of anomalous regions. Effective anomaly detection improves product reliability, customer satisfaction, and manufacturing efficiency.

Traditional image anomaly detection methods face significant limitations when applied to industrial applications. Statistical algorithms and manually designed feature methods struggle with complex visual patterns and show poor adaptation to varying environmental conditions^2^. These approaches often need extensive manual adjustments for thresholds and rules^3^, while lacking the pixel-level precision needed in manufacturing. Their slow processing also makes real-time detection difficult^4^.

Deep learning approaches have demonstrated improvements in anomaly detection performance compared to traditional methods. CNN-based methods, such as convolutional autoencoders and U-Net variants, have shown substantial improvements over traditional methods^5^. These networks excel at learning hierarchical visual features and can handle complex texture patterns effectively. However, CNN methods face inherent limitations due to their local receptive fields, which can miss long-range spatial dependencies crucial for understanding global image context.

Transformer-based architectures have recently gained attention in computer vision tasks, including anomaly detection^6^. Vision Transformers can capture global relationships through self-attention mechanisms, potentially addressing CNN limitations. Recent research has explored various Transformer-based approaches for video anomaly detection^7,8^ and efficient spatiotemporal feature fusion methods^9^, demonstrating the growing interest in advanced architectures for anomaly detection across different domains. Nevertheless, standard Transformers require substantial computational resources and often struggle with fine-grained local features that are critical for detecting small manufacturing defects. However, standard Transformers require substantial computational resources and often struggle with fine-grained local features that are critical to detecting small manufacturing defects. Recent research has explored various advanced architectures including attention-based methods^10,11^, enhanced autoencoders^12–14^, variational approaches^15^, and Transformer-based frameworks^7,8^ for anomaly detection across different domains, demonstrating the active development in this field.

A persistent challenge across all deep learning approaches is the data imbalance inherent in industrial settings, where anomalous samples are rare and expensive to collect^16^. This constraint makes supervised training impractical and has driven research toward unsupervised and semi-supervised methodologies, though these typically achieve lower performance than their supervised counterparts.

Reconstruction-based methods have been widely adopted by using neural networks to learn normal image patterns^17,18^. These approaches detect anomalies by measuring reconstruction errors. Autoencoder or GAN-based models reconstruct normal images and identify anomalies when reconstruction fails^19^. However, their strong generalization ability sometimes reconstructs anomalous regions too well, reducing detection effectiveness. To address this, some methods use image inpainting for anomaly detection^20–22^. These techniques mask parts of normal images during training to develop repair capabilities. During testing, they use masks to create reconstructed images for comparison. Recent methods like DRAEM^23^ have explored synthetic anomaly generation but still face limitations with domain transfer and small anomaly detection in industrial environments.

To address these limitations, we propose SRDAD (Swin Transformer-Based Hybrid Reconstruction Discriminative Network), an integrated architecture for industrial anomaly detection. The method combines reconstruction and discrimination approaches through three main components. First, a natural anomaly image generation module produces simulated defects that closely resemble real-world anomalies. Second, a Swin-UNet reconstruction subnetwork enables efficient image reconstruction. Third, a contrastive discrimination subnetwork facilitates end-to-end detection and localization of anomalous regions. Experiments on the MVTec AD benchmark dataset demonstrate the effectiveness of the proposed method. The approach achieves 98.6% detection accuracy and 98.0% localization precision.

The rest of this paper is organized as follows. Section 1 elaborates on the related work pertinent to this study. Section 2 describes the proposed SRDAD algorithm. Section 3 presents the experimental verification and analysis. Finally, Section 4 concludes with this research.

## Related work

This section examines the key techniques and methodologies that form the foundation of the proposed approach, focusing primarily on reconstruction-based anomaly detection algorithms, transformer-based anomaly detection algorithms, and visual transformer architectures.

### Reconstruction-based anomaly detection methods

Reconstruction-based methods can be categorized into several approaches based on their core technical principles. These methods rely on the fundamental assumption that models trained exclusively on normal samples will struggle to accurately reconstruct anomalous regions. This enables anomaly detection and localization by analyzing differences between the input and reconstructed images. Early approaches implemented various architectures including autoencoders, variational autoencoders (VAEs)^24^, and generative adversarial networks (GANs)^25^. However, these methods faced a significant challenge: the powerful generalization capabilities of deep neural networks often allowed successful reconstruction of anomalous regions, undermining detection performance. This limitation prompted researchers to develop specialized techniques to constrain model generalization.

Memory-enhanced reconstruction methods address the generalization problem by incorporating external memory structures. These approaches create feature repositories during training using normal samples, storing diverse features representing distinct normal patterns. During inference, reconstruction occurs by retrieving the most similar features from the repository for each region of the image. This approach effectively reduces the unwanted reconstruction of anomalous regions. Park et al. proposed a memory-guided normality approach for anomaly detection^26^. Further studies by Hou et al. developed multiscale memory architectures to address granularity limitations^27^.

Inpainting and simulation-based reconstruction methods represent another important category. Inpainting techniques train models to restore artificially masked regions to their normal appearance, enhancing contextual information learning. Yan et al. proposed a semantic context-based framework that uses masked portions of normal images to train reconstruction models^28^. Zavrtanik et al. initially divided images into $k \times k$ grids, randomly splitting them into multiple subsets with masks to leverage contextual information for restoration^29^. Building on this work, they later developed DRAEM^23^, which implements a defect simulation methodology that generates diverse anomalous samples with corresponding segmentation maps. This approach simultaneously learns representations of anomalous images and their normal reconstructions while establishing decision boundaries between normal and anomalous patterns. DRAEM enables direct anomaly localization without requiring complex post-processing. However, these methods often overfit synthetic anomaly patterns, limiting their generalization to real manufacturing defects. Recent work by Kim et al. has explored active learning approaches to improve classification performance through better data annotation strategies^30^. Although they focus on general classification tasks, the fundamental challenge of dealing with limited labeled data is common to computer vision applications including anomaly detection.

Reconstruction methods have evolved from simple autoencoders to more complex approaches. Early autoencoders often reconstructed anomalies too well, which led to memory-based methods that store normal patterns to constrain reconstruction. More recent approaches like DRAEM generate synthetic anomalies during training, which helps with localization but can struggle when synthetic patterns don’t match real defects well.

### Transformer-based methods for anomaly detection

Recent advances in deep learning have seen the emergence of Transformer architectures in anomaly detection. Traditional convolutional models typically struggle to capture global contextual information due to their inherent locality bias, potentially limiting their ability to detect complex anomaly patterns. Transformer-based methods leverage self-attention mechanisms to establish long-range dependencies, providing significant benefits for anomaly detection tasks.

The Vision Transformer (ViT)^31^ has shown impressive capabilities in computer vision by processing images as sequences of patches through self-attention mechanisms. Transformer-based models are particularly valuable for detecting subtle industrial defects that appear as contextual inconsistencies rather than obvious local abnormalities.

In industrial image anomaly detection, researchers have proposed several innovative transformer-based architectures. Jiang et al. introduced the Masked Swin Transformer Unet (MSTUnet) for industrial anomaly detection, leveraging the hierarchical structure of the Swin Transformer to extract multiscale features efficiently^32^. This architecture shows superior performance in detecting subtle defects. Similarly, Cai et al. proposed ITran, a transformer-based approach that addresses challenges in the evaluation of feature distribution and data scarcity^33^. By incorporating inductive bias and convolution operations, ITran reduces computational demands while achieving leading detection performance.

Hybrid approaches that combine transformer architectures with other methodologies have shown promising results^34^. Despite their advantages, transformer-based anomaly detection methods face challenges that include computational complexity, sensitivity to parameter settings, and requirements for substantial training data.

Transformers handle global patterns well but can miss local details that CNNs catch easily. CNNs work well for local texture problems but don’t see the bigger picture as effectively. This creates different strengths and weaknesses between the two approaches.

### Swin transformer for vision tasks

Several adaptations of the Vision Transformer have emerged to address limitations in the original architecture. The Swin Transformer^35^, which forms the foundation of the proposed method, introduces a hierarchical structure with shifted window attention mechanisms. This design effectively balances computational efficiency with receptive field coverage. By progressively merging patches and increasing feature dimensions across stages, the Swin Transformer captures multiscale representations essential for precise anomaly localization.

Existing Transformer-based anomaly detection methods primarily use standard ViT architectures within autoencoder frameworks^36^. While these approaches improve upon CNN-based methods, they often encounter challenges including high computational costs and difficulties processing high-resolution images. Additionally, most existing approaches fail to effectively leverage the hierarchical multi-scale features that Swin Transformers can provide, limiting their anomaly localization precision.

Swin Transformers process images in stages and use windowed attention, which makes them more efficient than standard Vision Transformers. They can also work with different scales better. However, most anomaly detection work with Swin Transformers hasn’t fully used these capabilities, especially for combining different types of features.

Existing synthetic anomaly generation methods often rely on external textures that may not adequately represent target object characteristics, creating domain gaps that limit practical effectiveness. Most approaches employ single paradigms-either reconstruction for pattern deviation detection or discrimination for precise localization-without exploring their potential integration. While transformer-based methods have demonstrated global context modeling capabilities, their application in industrial settings has primarily utilized standard architectures within autoencoder frameworks, potentially underutilizing hierarchical multi-scale features essential for diverse defect analysis. This work addresses these challenges by proposing SRDAD, which integrates natural anomaly generation with hybrid reconstruction-discrimination training and hierarchical feature processing for enhanced industrial anomaly detection.

## SRDAD for anomaly detection

**Fig. 1:**
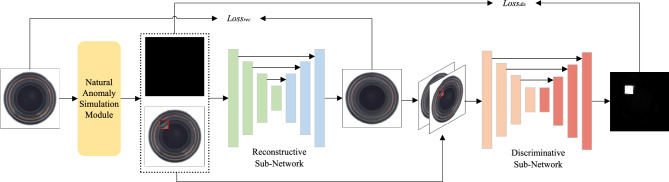
The overall architecture of SRDAD. The framework contains a Natural Anomaly Generation Module, a multi-level Reconstructive Sub-Network, and a Discriminative Sub-Network for pixel-level anomaly mapping, with interconnections optimizing both detection and localization.

The overall framework of SRDAD is shown in Figure 1. The architecture processes input images through three sequential components: the natural anomaly generation module creates synthetic defects from normal images, the Swin-UNet reconstruction network attempts to restore images to normal appearance, and the discrimination network identifies anomalous pixels. The two loss functions Loss_rec and Loss_dis enable joint optimization of both reconstruction and discrimination. It integrates three complementary components into a unified framework: a natural anomaly image generation module, a Swin Transformer-based reconstruction subnetwork, and a CNN-based recognition subnetwork. This architecture addresses key limitations of existing methods through targeted innovations in each component. By generating anomalies directly from the target object, this approach enhances the generalization ability of the model without requiring additional external datasets. The reconstruction subnetwork leverages the hierarchical structure of the Swin Transformer to simultaneously model global context and local details, while the segmentation subnetwork integrates specialized modules to optimize anomaly discrimination.

### Natural anomaly simulation module

To address the visual and distributional discrepancies between DRAEM-generated anomalies and real industrial defects, our module implements a physically constrained generation process with three key stages. Unlike existing methods that introduce external textures or random noise, our approach generates anomalies exclusively from patches extracted from the target object itself, ensuring that synthetic defects maintain the visual characteristics and material properties of the original object. Transformation parameters are calibrated based on statistical analysis of real defect datasets, constraining rotation angles, and scaling factors within ranges observed in actual production environments.

#### Target region extraction

The process begins with the precise localization of the target objects. For an input image $I \in \mathbb {R}^{H\times W\times 3}$, we first compute an adaptive threshold using Otsu’s method to account for varying illumination conditions:

1 $$\begin{aligned} B(x,y) = \mathbb {I}(I(x,y)> \tau _{Otsu}) \end{aligned}$$

where $\tau _{Otsu}$ automatically adapts to the image intensity histogram. Then a morphological closing operation with $3\times 3$ square structuring element *k* is applied to smooth the boundaries and fill the small holes:

2 $$\begin{aligned} M = B \circ k, \quad k \in \{0,1\}^{3\times 3} \end{aligned}$$

**Fig. 2:**
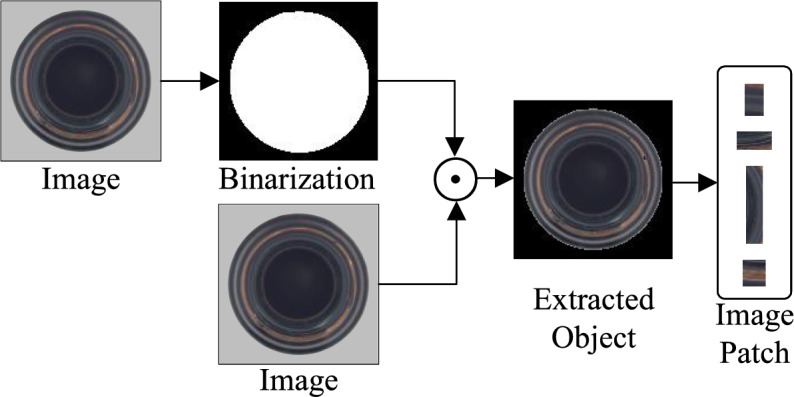
Process of Image Patch Extraction: original image undergoes binarization to create a mask, which is applied to the original image to extract the object, followed by patch sampling from the extracted region.

This produces the final target mask *M* that accurately covers the objects of interest while excluding the background regions. As shown in Figure 2, extracting image patches ensures that the extracted fragments come from the object itself.

#### Patch sampling and augmentation

From the target region $\{(x,y)|M(x,y)=1\}$, we randomly sample *N* image patches $\{P_i\}_{i=1}^N$ with sizes drawn from the empirical defect size distribution $p_{defect}(d)$ obtained through statistical analysis of real defect datasets. Each patch undergoes two levels of transformations:

1. **Geometric transformations** $T_{geom}$ simulate shape variations:
  3 $$\begin{aligned} T_{geom} \in \{\text {Rotation } \theta , \text {Scaling } s\}, \quad \theta \sim U(0,2\pi ), s \sim U(0.8,1.2) \end{aligned}$$
2. **Photometric transformations** $T_{photo}$ mimic appearance changes:
  4 $$\begin{aligned} T_{photo} \in \{\text {Add noise } \sigma , \text {Adjust brightness } \Delta \}, \quad \sigma \sim N(0,0.05), \Delta \sim U(-0.2,0.2) \end{aligned}$$

The transformed patch ${\hat{P}}_i = T_{geom}(T_{photo}(P_i))$ preserves physical realism through parameter ranges calibrated from actual defect observations. For instance, the rotation angle $\theta$ and scaling factor *s* are constrained to values observed in real production line defects.

#### Constrained synthesis

The augmented patches ${\hat{P}}_i$ are integrated into the original image through Poisson blending, which solves the optimization problem:

5 $$\begin{aligned} \min _{I_{ano}} \Vert \nabla I_{ano} - \nabla {\hat{P}}_i\Vert ^2 + \lambda \Vert I_{ano} - I\Vert ^2_{\partial R} \end{aligned}$$

where $\lambda$ controls the trade-off between preserving the source patch characteristics (${\hat{P}}_i$) and maintaining the target image (*I*) context. The boundary term $\partial R$ ensures seamless transitions along patch edges. Simultaneously, we generate the binary anomaly mask $Y \in \{0,1\}^{H\times W}$ that precisely marks synthetic defect regions for training supervision.

**Fig. 3:**
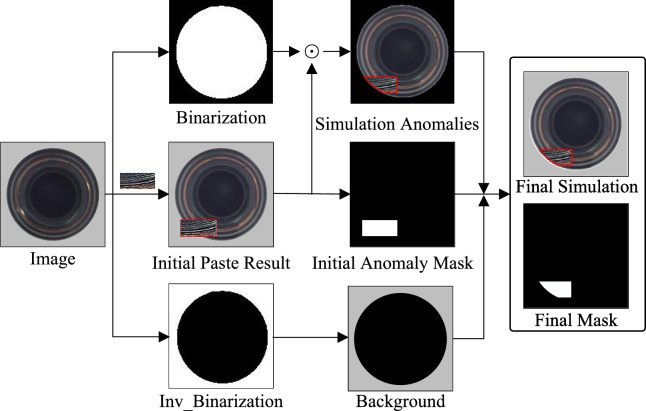
Process of Generating Simulated Anomalies and Their Corresponding Annotations.

Figure 3 illustrates the process for generating simulated anomaly images with their corresponding annotations. The process begins with random patch placement on original images, which can create unrealistic effects when patches extend beyond object boundaries. To deal with this problem, we apply morphological operations with optimized thresholds to create mask and inverse mask images. These masks are then used to constrain anomalies within object boundaries through pixel-wise multiplication. The process preserves the original image background and combines it with the simulated anomalous objects to create the final composite images. Multiple iterations of this patching process can be applied to increase the diversity and complexity of the anomalies generated. Algorithm 1 summarizes the complete process of generating natural anomalies.

**Algorithm 1:**
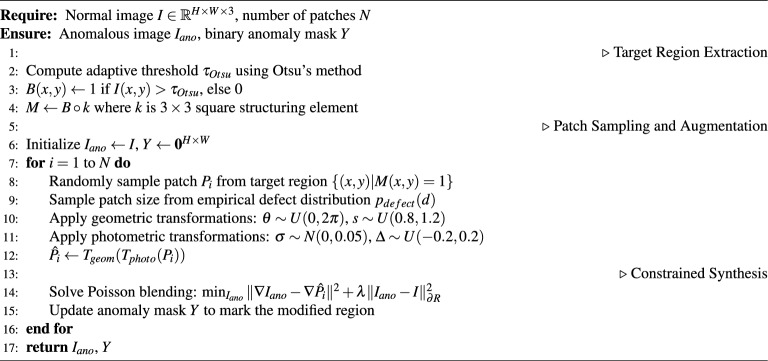
Natural Anomaly Generation Module

### Reconstructive subnetwork

**Fig. 4:**
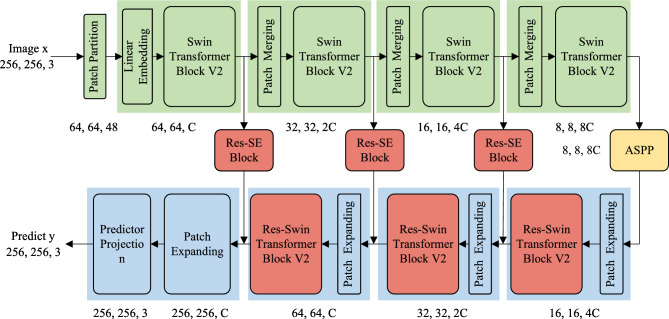
Comprehensive architecture of the reconstruction subnetwork.

The reconstructive subnetwork is a core component designed to restore anomalous images to their normal conditions. As illustrated in Figure 4, this network adopts a symmetric encoder-decoder architecture based on Swin-Unet^37^, specifically tailored for processing $256\times 256\times 3$ input images generated by our anomaly simulation module. The reconstruction procedure jointly operates through three coordinated mechanisms: multi-scale feature extraction via the encoder, hierarchical image restoration via the decoder, and cross-level feature preservation utilizing skip connections.

#### Encoder pathway

The encoder decomposes the input image into fundamental visual features. Using non-overlapping $4\times 4$ patches, the encoder transforms local image regions into a 96-dimensional embedding feature space through linear projection, formulated as follows:

6 $$\begin{aligned} E_i = WP_i + b \end{aligned}$$

where $E_i$ denotes the embedded feature vector corresponding to the *i*-th image patch, $W \in \mathbb {R}^{96\times 48}$ is a learnable weight matrix, $P_i \in \mathbb {R}^{4\times 4\times 3}$ is the input image patch, and $b \in \mathbb {R}^{96}$ is the bias term.

These embedded features subsequently undergo refinement through multiple Swin Transformer blocks, which alternate between regular and shifted window-based self-attention to capture local and global contextual information. Between successive transformer stages, patch merging layers progressively reduce spatial resolution while increasing feature dimensionality, from initial $64\times 64\times 96$ to final $8\times 8\times 768$ at the deepest encoder level.

#### Multi-scale feature fusion

**Fig. 5:**
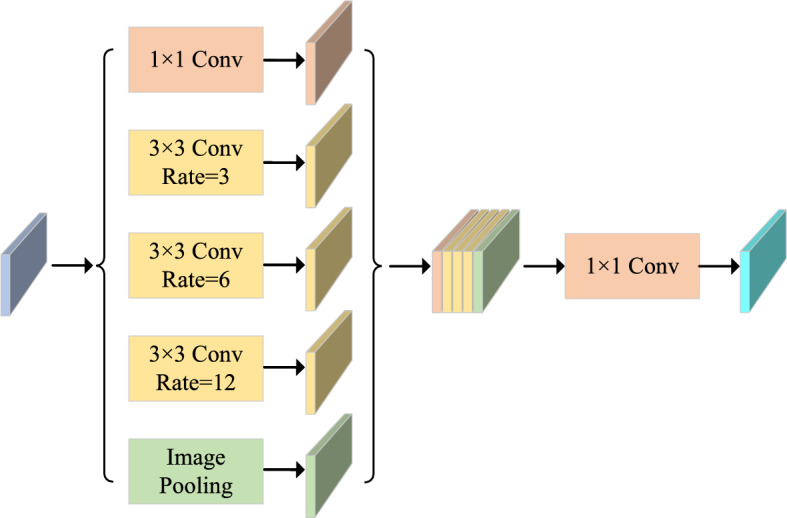
Detailed structure of the ASPP module. The module consists of three parallel dilated convolutional branches with dilation rates of 3, 6, and 12 respectively, alongside a global context branch implemented by average pooling. Feature maps from all branches are concatenated channel-wise to integrate fine details with global context.

At the deepest encoder stage, an Atrous Spatial Pyramid Pooling (ASPP) module aggregates multi-scale contextual information, as depicted in Figure 5. This module processes features through four parallel pathways, formally defined as:

7 $$\begin{aligned} F_{\text {ASPP}} = [F_3, F_{6}, F_{12}, \text {GAP}(X)] \end{aligned}$$

where $F_r$ represents the feature map obtained from dilated convolution with dilation rate *r* ($r \in \{3,6,12\}$), $\text {GAP}(X)$ denotes the globally averaged pooled features, and $[\cdot ]$ indicates the channel-wise concatenation operator.

This design leverages dilated convolutions for local detail preservation and average pooling for global context integration, thus enabling comprehensive multi-scale representations.

#### Decoder with residual learning

The decoder pathway reverses the encoder’s dimensionality reduction via a series of patch expansion layers. Each decoder block incorporates residual learning by establishing direct connections between inputs and outputs:

8 $$\begin{aligned} {\hat{X}}_l = \text {SwinBlock}(X_l) + WX_l \end{aligned}$$

where ${\hat{X}}_l$ represents the output features at decoder level *l*, $\text {SwinBlock}(\cdot )$ denotes the Swin Transformer block operation, and *W* is a learnable linear projection.

This residual design alleviates gradient vanishing and preserves feature fidelity throughout the upsampling procedure. The patch expansion layers progressively restore spatial resolution from $8\times 8\times 768$ back to the original $256\times 256$, halving feature depth at each upsampling step.

#### Feature preservation via skip connections

**Fig. 6:**
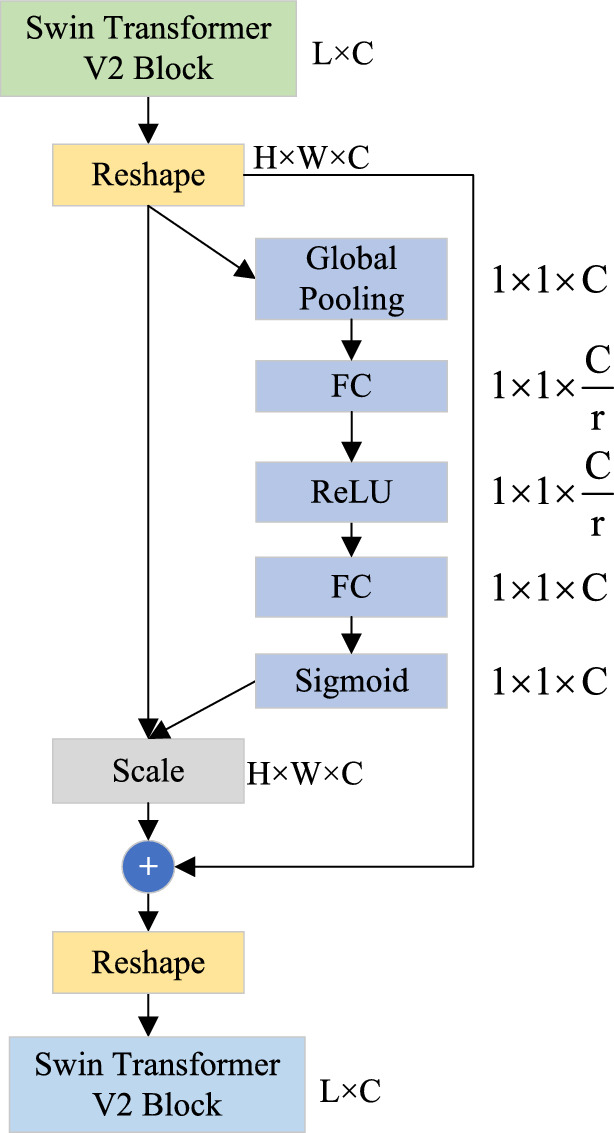
Residual attention mechanism for skip connections. The encoder features (left) and decoder features (right) are combined through an attention gate (center). The sigmoid activation generates spatial attention weights, selectively emphasizing encoder feature regions relevant for reconstruction.

Figure 6 illustrates the enhanced skip connection mechanism, which employs an attention-based gating strategy between corresponding encoder and decoder features:

9 $$\begin{aligned} F_{\text {skip}}^l = \sigma (W_1[E^l, D^l]) \odot (W_2E^l) \end{aligned}$$

where $F_{\text {skip}}^l$ denotes the gated feature map at level *l*, $\sigma$ is the sigmoid activation function, $\odot$ indicates element-wise multiplication, $W_1$ and $W_2$ are learnable weight matrices, and $[E^l, D^l]$ denotes concatenated encoder-decoder features.

This gating mechanism selectively preserves relevant context while suppressing redundant information during image reconstruction.

The final reconstructed image aggregates processed decoder features and refined skip connection features via a linear projection:

10 $$\begin{aligned} {\hat{I}}_{\text {normal}} = \text {Linear}\left( \sum _{l=1}^4 F_{\text {skip}}^l + \text {Decoder}(F_{\text {ASPP}})\right) \end{aligned}$$

where $\text {Linear}(\cdot )$ represents the final projection, $\sum _{l=1}^4 F_{\text {skip}}^l$ aggregates skip features across all levels, and $\text {Decoder}(F_{\text {ASPP}})$ is the decoder output feature map.

### Discriminative subnetwork

To further enhance detection and localization capabilities, this study replaces the traditional pixel-wise differencing approach commonly employed in reconstruction-based anomaly detection models. Inspired by the DRAEM model, a dedicated discriminative subnetwork is proposed for SRDAD, serving as a classifier to achieve end-to-end anomaly detection and localization without complex post-processing. This network adopts a convolution-based Unet architecture enhanced by Res-SE modules and the Channel Cross-fusion Transformer (CCT) structure to improve segmentation accuracy and localization precision. The discriminative approach leverages the observation that reconstruction quality varies systematically between normal and anomalous regions. While normal areas typically exhibit consistent reconstruction fidelity, anomalous regions display characteristic reconstruction deviations that can be learned and exploited for classification purposes.

**Fig. 7:**
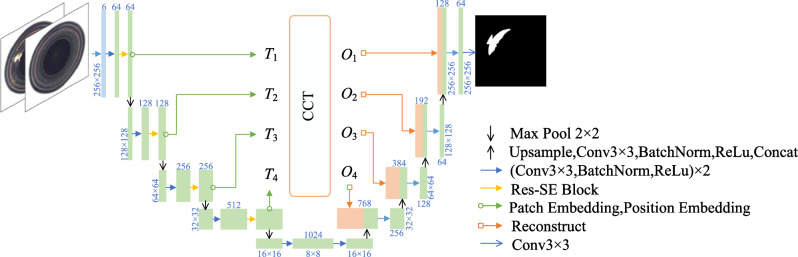
Structure of Skip-Connection with Residual Attention.

The discriminative subnetwork takes as input the channel-wise concatenation of anomalous and reconstructed images, outputting a predicted anomaly map $M_{\text {pre}}$, where pixels labeled as 0 represent normal regions and 1 represent anomalies. As depicted in Figure 7, Res-SE modules are integrated into each encoder downsampling layer to highlight significant features and suppress irrelevant information

**Fig. 8:**
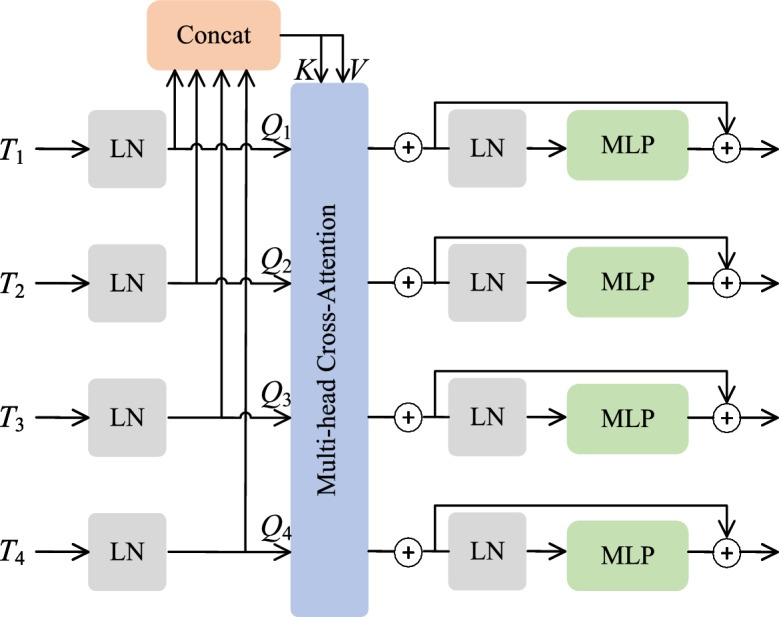
Architecture of Transformer with Channel Cross-fusion (CCT).

Moreover, the original skip connections of the Unet are replaced by the CCT structure (illustrated in Figure 8), which captures complex channel dependencies and reduces semantic gaps between encoder and decoder. The CCT employs multi-scale embeddings and multi-head channel cross-attention (MCA), formally defined as follows:

Firstly, the multi-scale encoder features are reshaped and tokenized into four scale tokens $T_i (i=1,2,3,4)$. These tokens are utilized as queries $Q_i$, and their concatenation $T_\Sigma$ serves as key $K$ and value $V$:

11 $$\begin{aligned} Q_i = T_i W_{Q_i}, \quad K = T_{\Sigma } W_K, \quad V = T_{\Sigma } W_V \end{aligned}$$

where $W_{Q_i} \in \mathbb {R}^{C_i \times d}$, $W_K \in \mathbb {R}^{C_{\Sigma } \times d}$, and $W_V \in \mathbb {R}^{C_{\Sigma } \times d}$ denote the corresponding learnable projection weight matrices. The parameter $d$ signifies the embedding dimension, whereas $C_i$ represents the channel dimensions of the four feature maps, and $C_{\Sigma }$ is the channel dimension after concatenation.

Then, the multi-head channel cross-attention (MCA) is computed as:

12 $$\begin{aligned} CA_i = \text {softmax}\left( \frac{Q_i K^T}{\sqrt{d}}\right) V \end{aligned}$$

Given multiple attention heads, the output of multi-head cross-attention is the average of all heads:

13 $$\begin{aligned} MCA_i = \frac{1}{N}\sum _{n=1}^{N} CA_i^{(n)} \end{aligned}$$

where $N$ is the number of attention heads. MLP operations and residual connections then yield the output features $O_i$:

14 $$\begin{aligned} O_i = MCA_i + \text {MLP}(Q_i + MCA_i) \end{aligned}$$

To simplify notation, the normalization layers are omitted from the above formulation. In our experiments, $N$ is set to 4, and a four-layer CCT structure is constructed, resulting in four outputs $O_i (i=1,2,3,4)$ corresponding to the encoder layers. These outputs are individually upsampled and reconstructed through convolutional layers, and then concatenated with the corresponding decoder layer features for further feature fusion. Consequently, the decoder effectively leverages multi-scale encoder features, enriching semantic content for anomaly localization.

To reduce noise from $M_{\text {pre}}$, a convolution operation with a mean filter $f_{s\times s}$ is applied, and the global maximum value of the resulting feature map is selected as the final image-level anomaly score ($\text {score}$):

15 $$\begin{aligned} \text {score} = \max (M_{\text {pre}} * f_{s\times s}) \end{aligned}$$

where $f_{s\times s}$ denotes a mean filter kernel of size $s\times s$, and $*$ represents the convolution operator.

### Loss function

The proposed SRDAD is a hybrid network consisting of reconstruction and discriminative subnetworks; thus, the overall loss function comprises two corresponding components. For the reconstruction subnetwork, a combination of Mean Square Error (MSE) and Structural Similarity Index Measure (SSIM) losses is employed. The MSE loss quantifies pixel-level differences between the original and reconstructed images:

16 $$\begin{aligned} \text {Loss}_{\text {MSE}} = \frac{1}{N} \sum _{i=1}^{N} (x_i - {\hat{x}}_i)^2 \end{aligned}$$

where $x_i$ and ${\hat{x}}_i$ denote the pixel values of the input and reconstructed patches respectively, and $N$ is the total number of pixels in the masked image patch. Additionally, SSIM loss incorporates perceptual similarity, aligning the reconstruction closer to human visual perception:

17 $$\begin{aligned} \text {SSIM}(x,y) = \frac{(2{\bar{x}}{\bar{y}} + C_1)(2S_{xy} + C_2)}{({\bar{x}}^2 + {\bar{y}}^2 + C_1)(S_x^2 + S_y^2 + C_2)} \end{aligned}$$

where ${\bar{x}}, {\bar{y}}$ represent the mean values of images $x$ and $y$, $S_x^2, S_y^2$ denote their respective variances, $S_{xy}$ is their covariance, and $C_1, C_2$ are constants ensuring numerical stability. The total reconstruction loss is thus defined as:

18 $$\begin{aligned} \text {Loss}_{\text {rec}} = \alpha \text {Loss}_{\text {MSE}} + \beta (1 - \text {SSIM}) \end{aligned}$$

where $\alpha$ and $\beta$ are weighting parameters balancing the two losses.

Considering the inherent imbalance between anomalous and normal regions in practical scenarios, the discriminative subnetwork utilizes Focal Loss^38^, addressing this imbalance by emphasizing difficult-to-classify samples:

19 $$\begin{aligned} \text {Loss}_{\text {focal}} = - \alpha _t (1 - p_t)^\gamma \log (p_t) \end{aligned}$$

where $p_t$ denotes the predicted probability, $\alpha _t$ represents the weighting factor balancing positive and negative samples, and $\gamma$ is the focusing parameter, typically set to 2.

Finally, the overall loss function for training SRDAD is defined as the summation of both reconstruction and discriminative losses:

20 $$\begin{aligned} \text {Loss}_{\text {SRDAD}} = \text {Loss}_{\text {rec}} + \text {Loss}_{\text {focal}} \end{aligned}$$

### Complexity analysis

The complexity of SRDAD encompasses both computational demands and parameter requirements, which are essential considerations for industrial deployment. For an input image of resolution $H \times W$, the natural anomaly simulation module operates with a time complexity of ${\mathcal {O}}(H \times W + N \times P^2 \times \log (P))$, where *N* represents the number of sampled patches and *P* denotes the average patch size. The Poisson blending operation contributes significantly to this complexity but ensures seamless anomaly integration.

The reconstructive subnetwork, based on Swin-Unet architecture, represents the computational core of SRDAD. Its time complexity is dominated by the Swin Transformer operations at ${\mathcal {O}}(L \times M \times W_s^2 \times C + L \times H \times W \times C^2)$, where *L* is the total number of transformer layers, *M* is the number of windows, $W_s$ is the window size, and *C* represents the channel dimension. The ASPP module, while adding computational overhead, enables multi-scale feature fusion critical for detecting anomalies of varying sizes. The discriminative subnetwork, with its CNN-based Unet structure enhanced by Res-SE and CCT components, contributes a complexity of ${\mathcal {O}}(H \times W \times C^2 + C^2)$. The space complexity primarily involves storage for model parameters at ${\mathcal {O}}((L_1+L_2) \times C^2 + C^2)$ and feature maps during processing at ${\mathcal {O}}(H \times W \times C)$.

Parameter analysis reveals that SRDAD contains approximately 47.2 million parameters in total, with the reconstructive subnetwork accounting for 19.4 million and the discriminative subnetwork containing 27.8 million. This parameter count significantly exceeds that of comparable models such as DRAEM (26.5 million) and InTra (23.7 million). The increased parameter count stems primarily from the Swin Transformer blocks in the reconstructive network and the enhanced CCT structure in the discriminative network.

Despite its higher computational requirements, SRDAD achieves meaningful performance gains, with a 0.6% improvement in detection accuracy and a 0.7% improvement in localization precision compared to DRAEM. In practical terms, SRDAD processes a 256$\times$256 resolution image in approximately 85 milliseconds on an NVIDIA TITAN Xp GPU, making it suitable for many industrial inspection scenarios. However, for applications with more stringent real-time requirements, future optimization through techniques such as model pruning and knowledge distillation could maintain performance while reducing complexity. This performance-complexity trade-off underscores the value of SRDAD in high-precision industrial anomaly detection applications where detection accuracy justifies the additional computational investment.

## Experiments

This section presents a comprehensive experimental evaluation of our proposed method, including experimental setup, comparative experiments, and ablation studies.

### Experimental setup and datasets

The hardware and software settings used in our experiments are presented in Table 1. All experiments were conducted on a workstation with Intel(R) Core i7-8700K CPU, NVIDIA TITAN Xp GPU, and 32GB memory. The software environment consisted of Ubuntu 20.04.4 LTS operating system, PyTorch 1.13.0 as the deep learning framework, and Python 3.8.16 running on Anaconda 3 for package management and environment control.

**Table 1:** Hardware and Software Configuration.

**Hardware Configuration**	
CPU	Intel(R) Core i7-8700K @ 3.70GHz
GPU	NVIDIA TITAN Xp 12 G
Memory	32GB
Operating System	Ubuntu 20.04.4 LTS
Deep Learning Framework	PyTorch 1.13.0
Development Environment	Anaconda 3, Python 3.8.16

The dataset used in this experiment is MVTec-AD^39^, a widely used benchmark for unsupervised industrial anomaly detection. MVTec-AD comprises 15 different object and texture categories, with normal images for training and both normal and anomalous images for testing, accompanied by pixel-level annotations. The dataset features realistic scenes, diverse anomalies, and precise annotations, making it ideal for evaluating anomaly detection methods.

In the case of multi-class classification, accuracy measures the proportion of correct classifications and the total number of classified terms. In the process of anomaly detection for industrial images, to evaluate the effectiveness of the model, this paper conducted qualitative and quantitative analysis of the detection results by calculating Area Under the Receiver Operating Characteristic curve(AUROC). The formula is as follows:

21 $$\begin{aligned} AUROC = \int _0^1 \text {TPR} \, d(\text {FPR}) \end{aligned}$$

The AUROC evaluation metric involves a comprehensive consideration of both the true positive rate and false positive rate of the detection results. The actual physical meaning is the probability of randomly selecting a pair of positive and negative samples and judging that the positive sample is ranked before the negative sample. The larger the AUROC, the better the detection effect, indicating that the positive sample is ranked before the negative sample. In addition, the localization AUROC score was used to measure the defect localization effect of the model, and ultimately the detection AUROC score was used as the anomaly detection score to determine the abnormality of the sample.

During the experiment, the original high-resolution images were first uniformly resized to 256$\times$256 pixels to ensure the consistency of the input data. Image augmentation was performed using Python’s ImgAug library during the simulated anomaly image process. For each object type, training was conducted over 500 iterations with a batch size set at 8. The weight coefficient for the reconstruction subnetwork loss function, $\alpha$, was set to 0.9, while the weight coefficient, $\beta$, was set to 0.1. The initial learning rate was established at 0.0001 and was multiplied by 0.1 after reaching the 400th iteration, with the AdamW optimizer used for optimizing model parameters.

### Comparative experiments

In the current study, the proposed SRDAD model is juxtaposed with analogous image anomaly detection methods such as RIAD^29^, InTra^40^, CutPast^41^, Natural Synthetic Anomalies(NSA)^42^, and DRAEM. DRAEM has been delineated in a preceding section, whereas the core technological approaches of the other methods are expounded as follows:

**Table 2:** Image-level auroc anomaly detection comparative results (%).

Class		RIAD	InTra	CutPast	NSA	DRAEM	**SRDAD**
Texture	Carpet	84.2	98.8	93.1	95.6	97	98.9
	Grid	99.6	100	99.9	99.9	99.9	100
	Leather	100	100	100	99.9	100	100
	Tile	98.7	98.2	93.4	100	99.6	99.4
	Wood	93	97.5	98.6	97.5	99.1	99.7
	Average	95.1	98.9	97	98.6	99.1	99.6
Object	Bottle	99.9	100	98.3	97.7	99.2	100
	Cable	81.9	70.3	80.6	94.5	91.8	91.4
	Capsule	88.4	86.5	96.2	95.2	98.5	98.4
	Hazelnut	83.3	95.7	97.3	94.7	100	100
	Metalnut	88.5	96.9	99.3	98.7	98.7	100
	Pill	83.8	90.2	92.4	99.2	98.9	97.4
	Screw	84.5	95.7	86.3	90.2	93.9	98.6
	Toothbrush	100	100	98.3	100	100	100
	Transistor	90.9	95.8	95.5	95.1	93.1	96.3
	Zipper	98.1	99.4	99.4	99.8	100	100
	Average	89.9	93	94.3	96.5	97.4	98.2
Average All		91.7	95.0	95.2	97.2	98.0	98.6

Both RIAD and InTra harness the image repair strategy to detect anomalies within images. RIAD segmentizes the image into a grid system, obfuscating and subsequently reconstructing specific regions using a CNN-based U-Net architecture. However, it has been critiqued for its tendency towards excessive generalization. In contrast, InTra employs a Transformer to capture long-range semantic information, reconstructing concealed image segments by interpreting contextual data surrounding the obscured section, thereby mitigating the overgeneralization issues inherent in RIAD. The CutPaste approach endorses self-supervised learning, crafting pseudo-anomalous samples through cutting and pasting within the image frame to train a binary classifier, eliminating the requirement for genuine anomalous samples. NSA deploys the Poisson image editing technique to seamlessly generate naturally synthesized anomalous images by amalgamating scaled local regions from disparate images, though it imposes a more substantial computational demand. Collectively, these methodologies contribute valuable explorations and distinct solution strategies for advancing the field of image anomaly detection.

**Fig. 9:**
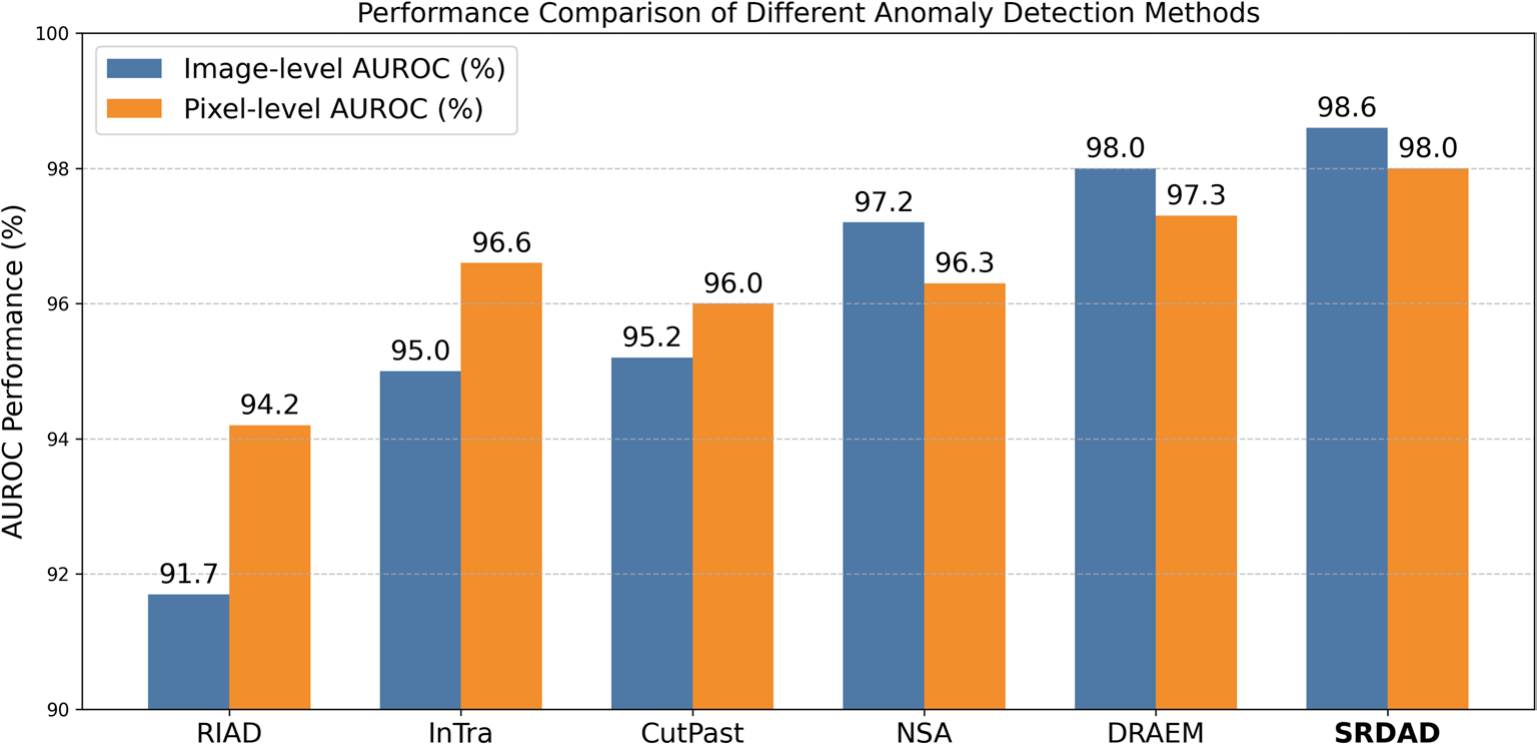
Performance comparison of different anomaly detection methods in terms of image-level and pixel-level AUROC on the MVTec AD dataset.

The experimental results of SRDAD are compared with the aforementioned methods, wherein the comparative method data are all derived from their respective original papers. Table 2 presents a comparative analysis of AUROC results for image-level anomaly detection, whereas Table 3 delineates the AUROC outcomes for pixel-level anomaly localization, based on the findings. The visualization results are shown in the Figure 9. As illustrated, SRDAD consistently outperforms other comparative methods on both image-level and pixel-level AUROC metrics.

Table 3 provides a quantitative comparison of anomaly detection performance between SRDAD and other methods. These detection results indicate that methods introducing simulated anomaly information generally outperform those that solely rely on image reconstruction. SRDAD exhibits a remarkable detection performance, achieving 100% recognition accuracy on seven objects - grid, leather, bottles, hazelnuts, metal nuts, toothbrushes, and zippers. When compared to the optimal model DRAEM, the average score for texture categories is higher by 0.5%, for object categories by 0.8%, with an overall increase of 0.6% to reach 98.6%. Compared to the SMAD model proposed in the previous chapter, the detection performance of SRDAD has significantly increased by 2.4%. This effectively proves that SRDAD greatly enhances the accuracy and reliability of anomaly detection, by effectively combining simulated anomaly information, image reconstruction, and discriminative training techniques.

**Table 3:** Pixel-level auroc anomaly detection comparative results(%).

Class		RIAD	InTra	CutPast	NSA	DRAEM	**SRDAD**
Texture	Carpet	96.3	99.2	98.3	95.5	95.5	98.3
	Grid	98.8	98.8	97.5	99.2	99.7	99.1
	Leather	99.4	99.5	99.5	99.5	98.6	99.5
	Tile	89.1	94.4	90.5	99.3	99.2	99.3
	Wood	85.8	88.7	95.5	90.7	96.4	97.4
	Average	93.9	96.1	96.3	96.8	97.9	98.7
Object	Bottle	98.4	97.1	97.6	98.3	99.1	99.3
	Cable	84.2	91	90	96	94.7	96.1
	Capsule	92.8	97.7	97.4	97.6	94.3	96.9
	Hazelnut	96.1	98.3	97.3	97.6	99.7	99.7
	Metalnut	92.5	93.3	93.1	98.4	99.5	99.6
	Pill	95.7	98.3	95.7	98.5	97.6	97.1
	Screw	98.8	99.5	96.7	96.5	97.6	98.5
	Toothbrush	98.9	98.9	98.1	94.9	98.1	98.3
	Transistor	87.7	96.1	93	88	90.9	92.7
	Zipper	97.8	99.2	99.3	94.2	98.8	98.9
	Average	94.3	96.9	95.8	96	97	97.7
Average All		94.2	96.6	96.0	96.3	97.3	98.0

From the anomaly localization comparison results listed in Table 3, it can be observed that the performance of methods incorporating simulated anomaly information is comparable to those that depend only on image reconstruction, with InTra even surpassing CutPaste and NSA. SRDAD continues to maintain a superior performance in anomaly localization. In terms of texture categories, the average anomaly localization score of SRDAD has improved by 0.8% compared to the optimal model DRAEM. Looking at object categories, the average anomaly localization score of SRDAD has increased by 0.7% over DRAEM. Overall, SRDAD reaches an average anomaly localization score of 98.0%, surpassing other comparative methods and modestly exceeding DRAEM by just over 0.7%. Synthesizing the localization results across 15 object categories, the SRDAD model demonstrates outstanding performance in anomaly localization.

**Fig. 10:**
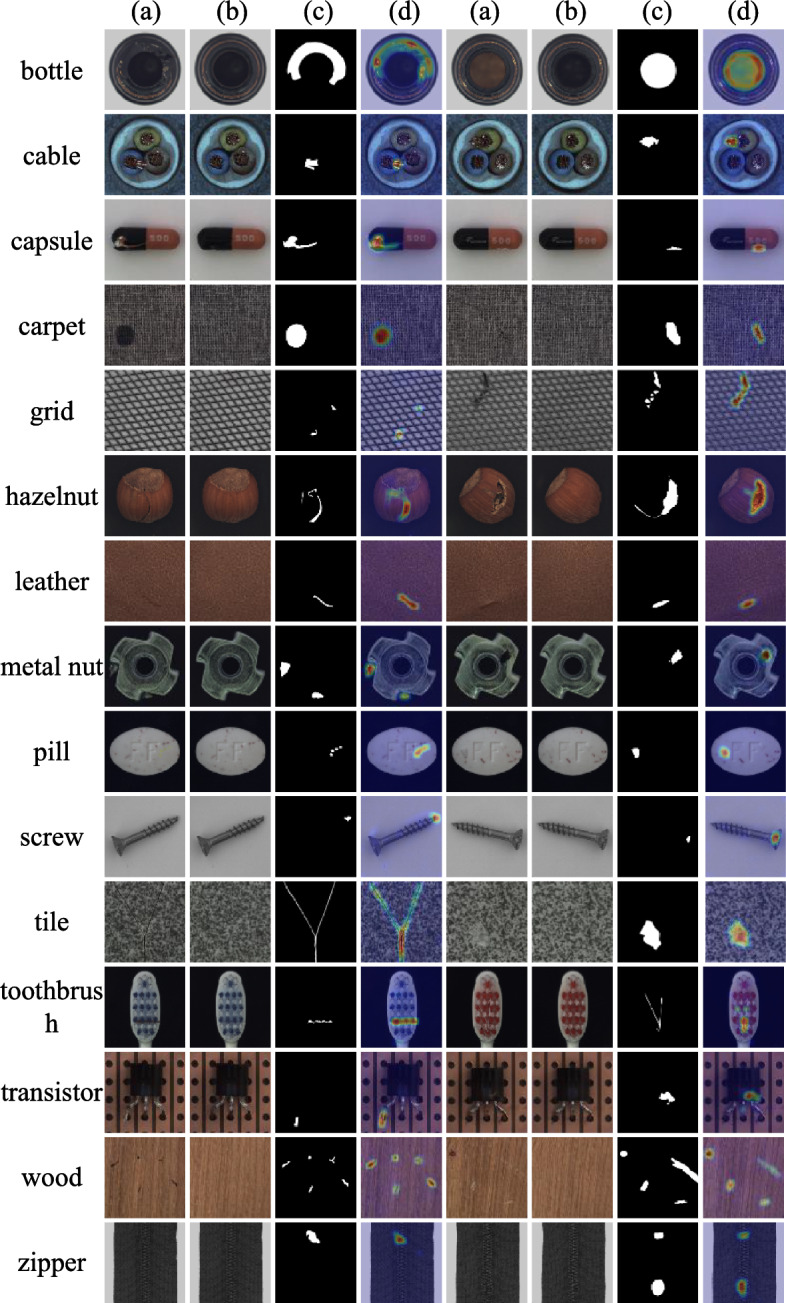
Detection samples from the MVTec-AD dataset. For each object category, four columns display: (a) original anomalous images, (b) SRDAD reconstructions, (c) ground-truth annotations, and (d) predicted anomaly heatmaps overlaid on original images.

To further illustrate the practical effects of SRDAD in anomaly detection and localization, visualized examples of the experimental results are shown in Figure 10. Two cases have been randomly selected for each object type, with each example comprising four images arranged in columns. Column ’a’ displays original anomaly images from different object test sets; column ’b’ presents the full reconstruction images obtained via SRDAD after training. Column ’c’ provides the ground-truth annotations corresponding to the anomaly images, while column ’d’ shows the predicted anomaly areas by the SRDAD model. The final column overlays the original image with an anomaly heat map, providing an intuitive visualization of the detection results. As demonstrated by the screw images, SRDAD is capable of accurately locating even minute anomaly areas.

###  Ablation experiments

Table 4 presents the ablation study results of the SRDAD model on the MVTec AD dataset, aiming to quantitatively evaluate the impact of each component on the model’s performance. In the table, “•” indicates that the corresponding module is used. In this set of experiments, the DRAEM model is used as the baseline, and its performance serves as the reference standard for the experiments. In the baseline model DRAEM, the reconstruction subnetwork is based on a CNN structure, while the discriminative subnetwork is based on a CNN’s Unet structure. DRAEM+ refers to integrating the Res-SE module and CCT structure into the discriminative subnetwork while maintaining the reconstruction subnetwork’s structure unchanged. Meanwhile, SRDAD-denotes the use of a pure Swin-Unet structure as the reconstruction subnetwork and a CNN’s Unet structure as the discriminative subnetwork, without additional modules. The final SRDAD model represents the integration of residual and pooling modules into the Swin-Unet structure, as well as the Res-SE module and CCT structure into the discriminative subnetwork. Through comparative analysis of the experimental results, it can be observed that the introduction of the Res-SE module and CCT structure has a good effect on anomaly localization. The use of Swin-Unet to construct the reconstruction subnetwork can enhance detection performance, and the integration of residual modules further contributes to improvement. With the combined final reconstruction-discriminative network, the SRDAD model achieves maximum performance.

**Table 4:** The ablation study results of the SRDAD model on the MVTec AD dataset, where Det means detection and Loc means Location.

Method	Reconstructive Subnetwork			Discriminative Subnetwork			Det(%)	Loc(%)
	CNN	Swin-Unet	Swin-Unet+	Unet	Res-SE	CCT		
DRAEM	•			•			98	97.3
DRAEM+	•			•	•	•	98.2	97.7
SRDAD-		•		•			98.3	97.4
SRDAD			•	•	•	•	98.6	98

**Fig. 11:**
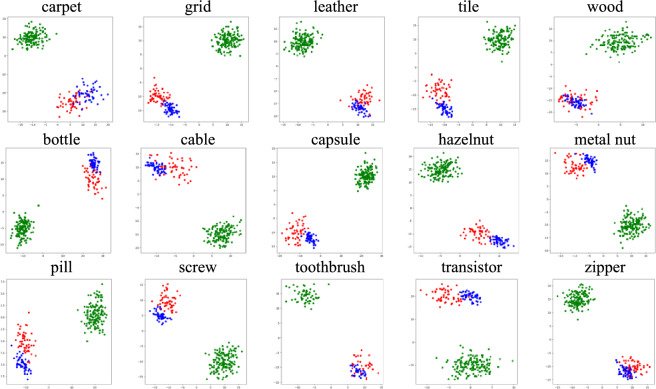
T-SNE Visualization Results, green dots represent normal sample, while red dots and blue dots represent real anomalous samples and anomalous samples simulated by the model.

To evaluate the effectiveness of the natural anomalous image generation module of the model, this study employed the t-distributed Stochastic Neighbor Embedding (t-SNE) algorithm for experimental validation^43^. To analyze the similarity in feature representation between the anomalous samples simulated by the model and real anomalous samples. The t-SNE algorithm is a powerful dimensionality reduction technique that can effectively transform high-dimensional data into two- or three-dimensional spaces for easy visual analysis. Experiments were conducted on the MVTec AD dataset, with 120 normal samples, 50 anomalous samples, and 50 simulated anomalous samples randomly selected for most objects. Due to the relatively small data volume for the toothbrush category, the number of samples was adjusted accordingly to 60 normal samples, 30 anomalous samples, and 30 simulated anomalous samples. These samples were input into the model, and their respective feature representations were extracted from the discriminative subnetwork. Subsequently, the t-SNE algorithm was utilized to generate a scatter plot as shown in Figure 11.

By analyzing these image results, it can be observed that the simulated anomalous samples cluster in similar regions in the feature space as the real anomalous samples, indicating that the simulated samples are effective and demonstrating the excellent anomalous simulation capability of the natural anomalous image generation module. This provides support for the training of the model. The results of the scatter plot also validate the model’s ability to effectively distinguish between normal and anomalous samples.

### Robustness and reconstruction capability evaluation

**Fig. 12:**
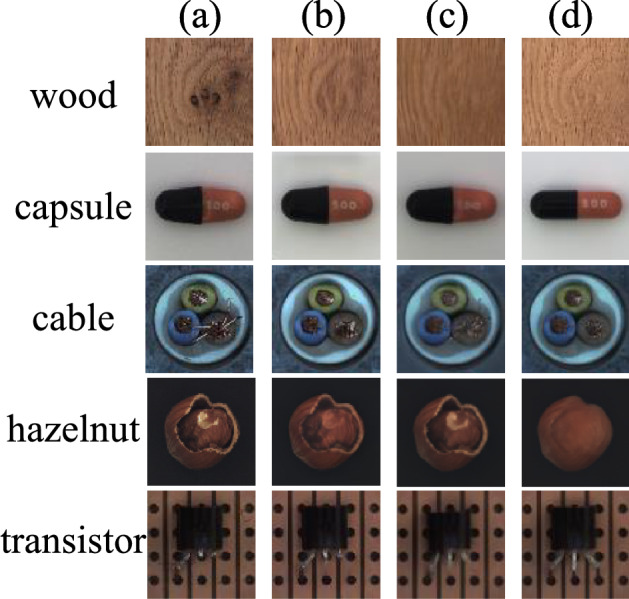
Visual comparison of reconstruction results for five object types.

In addition to ablation experiments, the reconstruction capabilities of the proposed SRDAD model were evaluated against DRAEM and InTra. Abnormal image samples were randomly selected from various object categories, and these models were used to reconstruct them. Figure 12 shows a visual comparison of reconstruction results for five object types. While DRAEM and InTra showed limitations in reconstructing abnormal regions to normal areas, SRDAD demonstrated superior reconstruction quality, particularly for larger anomalies. This was evident in the degree of correction and the accuracy of reconstruction details.

**Fig. 13:**
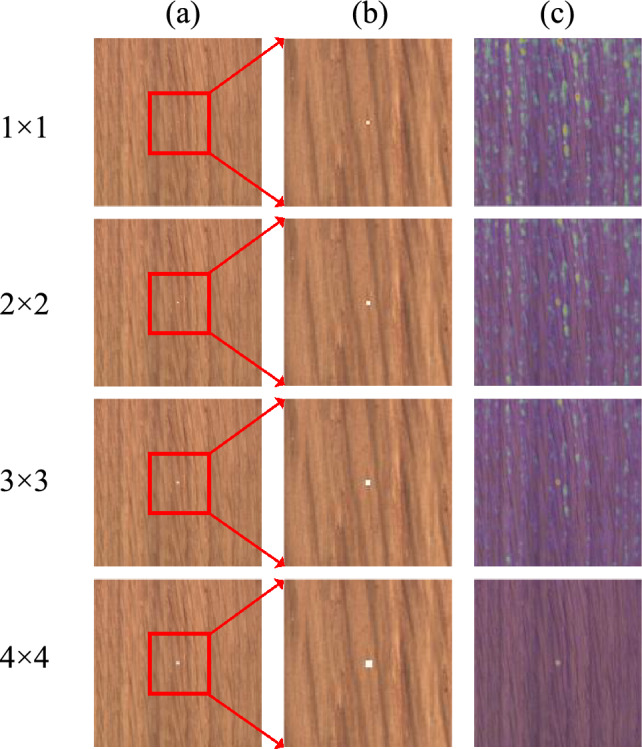
Simulation Results of Detecting Subtle Abnormalities.

Although an improvement in the detection of subtle abnormal regions was observed in the visualization results of SRDAD, to further ensure the model’s excellent detection and precise localization capabilities in detecting subtle abnormal regions, a quantitative experiment simulating subtle abnormalities was specifically conducted using wood as the detection object. Wood surfaces exhibit rich details, which on one hand makes it easier to present small-area abnormalities, and on the other hand, allows for testing the model’s discriminative ability in complex backgrounds. To accurately evaluate the model’s performance, the experiment artificially created simulated abnormal regions of different sizes and prominently labeled them in the center of the wood images. The experimental results are shown in Figure 13.

In the second column of images, four different sizes of abnormal regions highlighted with red boxes are displayed, with sizes of 1$\times$1, 2$\times$2, 3$\times$3, and 4$\times$4 abnormal pixel blocks, using a 256$\times$256-pixel original image as the background. From the experimental results, it can be seen that the model is already able to locate abnormal regions of 3$\times$3 pixels in size, and when the abnormal region expands to 4$\times$4 pixels, the model’s localization ability is significantly improved, fully demonstrating the model’s effective recognition and precise localization capabilities for small-area abnormalities.

To evaluate the robustness and noise resistance of the model under noisy conditions, toothbrushes and carpets were selected as experimental objects. To simulate the noise interference that images may encounter in the real world, image enhancement techniques were introduced to process the samples, including adding Gaussian noise and adjusting image parameters such as contrast, brightness, and blurriness.

**Fig. 14:**
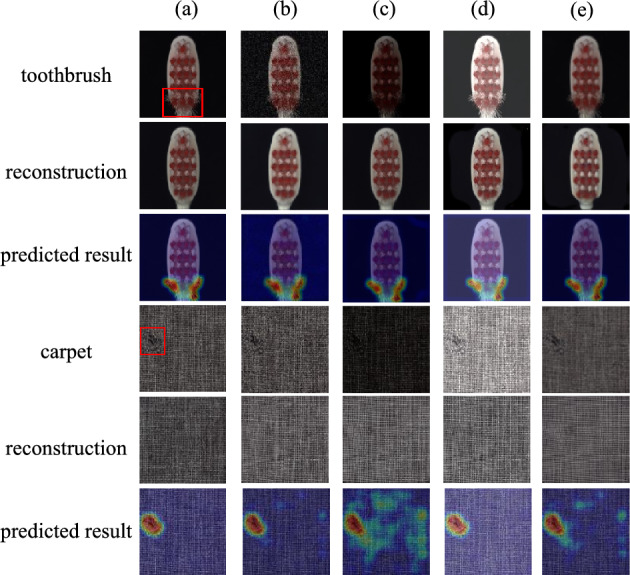
Results on the Noise Resistance of SRDAD.

As shown in Figure 14, the first row of each object in the experimental results exhibits the effects of applying different image enhancement techniques to the samples, thereby reflecting the impact of noise on image quality. Subsequently, these noise-affected images as well as the unprocessed original images were input into the SRDAD model for image reconstruction and anomaly localization. The reconstructed images and anomaly localization maps obtained are displayed in the second and third rows, respectively. Observing the experimental results, despite the significant impact of various noises on the quality of the sample images, the SRDAD model still demonstrated stable performance in reconstruction and localization, fully demonstrating its excellent noise resistance.

The experimental evaluation and method design are based on several assumptions. The methodology assumes that normal samples exhibit consistent patterns while anomalous regions deviate from these patterns. The method also assumes that normal training samples are sufficiently available, while labeled anomalous samples remain limited. The method further assumes that synthetic anomaly generation provides useful training information and that reconstruction and discrimination provide complementary detection capabilities. Due to its multi-component design, SRDAD involves slightly higher computational complexity than simpler methods. The method may require parameter adjustment to achieve optimal performance in different industrial sectors. Although better results were demonstrated on the MVTec AD, evaluation on additional datasets will strengthen the validation of the method.

## Conclusion

This paper proposes SRDAD, a hybrid framework that combines Swin Transformer-based reconstruction with contrastive discrimination for industrial anomaly detection. The approach addresses limitations of CNN-based methods by integrating a natural anomaly generation module that produces realistic synthetic defects, a Swin-UNet reconstruction subnetwork with enhanced residual connections for improved feature preservation, and a contrastive discrimination subnetwork with channel cross-fusion transformer architecture. This systematic combination leverages both global context modeling and local feature precision to improve detection performance across diverse anomaly types and scales.

Extensive experiments on the MVTec AD dataset demonstrate that SRDAD achieves 98.6% detection accuracy and 98.0% localization precision, representing improvements of 0.6% and 0.7% respectively over existing methods. The method shows particularly strong performance in challenging scenarios involving small anomalies and noisy environments. The method requires additional computational resources but represents a reasonable trade-off for high-precision applications. Ablation studies confirm the effectiveness of each architectural component.

SRDAD is well-suited for high-precision manufacturing applications including semiconductor inspection and precision electronics manufacturing. Future research directions include developing optimized variants through knowledge distillation and exploring adaptive anomaly generation techniques to extend effectiveness across broader industrial domains.

## Data Availability

The datasets used and/or analysed during the current study are available from the corresponding author on reasonable request.
